# Generation of a Double Reporter mES Cell Line to Simultaneously Trace the Generation of Retinal Progenitors and Photoreceptors

**DOI:** 10.3390/cells14040252

**Published:** 2025-02-10

**Authors:** Oleksandr Zabiegalov, Adeline Berger, Dhryata Kamdar, Kabirou Adamou, Chuanxi Tian, Martial Mbefo, Mathieu Quinodoz, Florian Udry, Carlo Rivolta, Corinne Kostic, Yvan Arsenijevic

**Affiliations:** 1 Unit of Retinal Degeneration and Regeneration, Department of Ophthalmology, University of Lausanne, 1004 Lausanne, Switzerland; chuanxi.tian@unil.ch (C.T.); martial.mbefo@chuv.ch (M.M.); fudry777@gmail.com (F.U.); 2 Unit of Epigenetics of Ocular Diseases, Department of Ophthalmology, University of Lausanne, 1004 Lausanne, Switzerland; adeline.berger@fa2.ch; 3 Ophthalmic Genetics Group, Institute of Molecular and Clinical Ophthalmology, 4031 Basel, Switzerland; dhryata.kamdar@iob.ch (D.K.); mathieu.quinodoz@iob.ch (M.Q.); carlo.rivolta@iob.ch (C.R.); 4 Department of Ophthalmology, University of Basel, 4031 Basel, Switzerland; 5 Group for Retinal Disorders Research, Department of Ophthalmology, University of Lausanne, 1004 Lausanne, Switzerland; kabirou.adamou@fa2.ch (K.A.); corinne.kostic@fa2.ch (C.K.)

**Keywords:** retinal organoids, reporter line, stem cells, retinal progenitor cells, *Rax* gene, cell tracing

## Abstract

Three-dimensional retinal culture systems help to understand eye development and the pathology of disorders. There is a need for reporter stem cell lines to allow in vitro studies on retinal progenitors and photoreceptors and their developmental dynamics or properties and to test therapeutic approaches. The isolation of pure progenitor populations or photoreceptor precursors may serve for drug, gene, and cell therapy development. Here, we generated a dual-reporter mouse embryonic stem cell line *Crx-GFP;Rax-mCherry* enabling the visualization or isolation of photoreceptors and retinal progenitors from retinal organoid settings. From day 4 organoids, we isolated mCherry-positive cells to assess their early retinal progenitor identity with proliferation tests as well as transcriptomic and proteomic profiling. The timing of eye field transcription factor expression at the transcriptomic and protein levels is in accordance with mouse retinogenesis. This new line will be helpful for rapidly investigating biological questions or testing therapeutics before using human induced pluripotent stem cells (iPSCs), which require a much longer time for retinal organoid formation.

## 1. Introduction

Visual input accounts for a significant part of a human’s world representation, and function loss or impairment thereof highly endangers the quality of life. Some of the most devastating degenerative eye diseases, such as dry age-related macular degeneration and inherited retinal dystrophies (IRDs), still lack treatment [1]. Animal models have been developed for decades to study eye development and devise new therapies, but species-specific differences push scientists to find alternative model systems. Ideally, a cellular model is required to dissect the biological and pathological features of a cell type. Since culturing photoreceptors is difficult and rarely successful [2], research studies in ophthalmology frequently use retinal explants as a source of retinal cells. Although this in vitro model is very relevant, it is difficult to handle in a homogenous manner when the experiment design necessitates more than 9–12 samples and implies the availability of numerous animals or human samples when different conditions and doses have to be tested. Appropriate transgenic mouse models are also needed when a specific gene function is analyzed. Consequently, optic-cup organoid technology has become more popular in the field, especially in studies of IRDs [3].

Organoids are 3D cellular structures generated from pluripotent or adult stem cells, progenitor cells, or cancer cells, able to recapitulate a specific organ compartment through self-organization after cell isolation or selection and spatially restricted lineage differentiation [4,5]. First derived in 2011 from mouse embryonic stem cells [6], mouse- and human-derived organoids have by now been produced for most tissue types representing a versatile tool for developmental and translational biology [7].

Several advantages make this system instrumental in studying retinal cells.

Firstly, all seven main retinal cell types appear during differentiation in a layered architecture [6,8]. Furthermore, the formation and maturation of the neural retina and RPE correspond to in vivo counterparts in temporal and spatial aspects, in particular, during the initial stages [9,10]. Additionally, some publications report that the mature photoreceptors form outer segments and respond to light [11,12,13,14,15].

That said, conversely, there are also serious limitations to this model. First, for example, the differentiation protocols are sensitive to the stem cell line used [12,16]. Second, the retinal pigmented epithelium is produced in most differentiation protocols but not juxtaposed to the organoid photoreceptor layer [7,17]. Third, the number of retinal ganglion cells is under-represented, likely due to low nutrition levels at their location and the lack of target brain regions for optic nerve outgrowth and survival [18,19]. Also, the absence of vascular system and immune cells hinders the modeling of certain conditions [20]. Finally, retinal organoids are heterogeneous, both within the same aggregate and between aggregates of the same experiment, which makes it challenging to perform standardized experiments [19]. However, regarding the latter, a recent publication offers a way to mitigate the heterogeneity problem [10].

To address some of these issues, it is crucial to trace and characterize early retinal progenitor cells (RPCs) at different time points in mouse retinal organoids at a molecular level, since all the retinal cell types are generated during neurogenesis. Their isolation may thus serve to study retinogenesis with natural RPCs during retina development [21], as previously documented, or to expand a pure population of retinal cells [22]. Additionally, detailed knowledge of RPC characteristics in the context of retinal organoids might help to facilitate their differentiation into photoreceptors, support the emergence of synaptic wiring between photosensitive, bipolar, and horizontal cells, and control photoreceptor maturation. Tracing of progenitors, cones, and rods can be used to study cell fate decisions and potentially aid in unraveling new molecular mechanisms of cell commitment for different retinal cell types [7].

In this study, we describe the generation of a mouse embryonic stem cell reporter line, which allows us to trace retinal progenitors at different developmental stages and follow photoreceptor differentiation. As a first step, an mCherry sequence was inserted by gene editing (CRISPR/Cas9^D10A^) after the *Rax* gene in a previously described mES cell line expressing GFP under the activation of *Crx* [23]. The *Rax* gene was selected for its critical role during eye field specification, retinal progenitor cell maintenance, and cell fate specification during retinogenesis [24]. Next, we characterized how one clone differentiates into retinal organoids with mCherry-positive cells starting to appear from day 4 after aggregation and differentiation. We performed profiling of early-born mCherry-positive cells using immunohistochemical staining. Transcriptomic and mass spectroscopy-based proteomic analysis of FACS-isolated cells allowed us to determine the lineage commitment of these cells corresponding to an early RPC. In perspective, these cells can serve as material for developmental studies and disease modeling, for example, to generate more homogeneous retinal organoids from early retinal progenitors or to better characterize the transition of RPC towards a differentiated state, including photoreceptors.

## 2. Materials and Methods

### 2.1. Mouse ES Cell Culture

For cell culture, we used a mouse embryonic stem cell (ES) line (*Crx-GFP* in a C57BL/6-129SvJ background) that was previously isolated and characterized in the lab [23] and a gene-edited line Crx-GFP;Rax-mCherry produced in this study from the former line. Before culturing them, cell culture plates (CLS3516, Corning, New York, NY, USA) were first coated with 0.2% gelatine (G1890, Sigma-Aldrich, Saint Louis, MO, USA) for at least 15 min at 37 °C, and, then, mouse embryonic fibroblasts (MEFs) were seeded on a plate, cultured for 3 passages in MEF medium (Table 1), and inactivated with mitomycin C (M0503-2MG, Sigma-Aldrich, Saint Louis, MO, USA) upon reaching confluency. The following day, ESCs were introduced on the monolayer of inactivated MEFs in an ESC culture medium (Table 2 and Table 3), supplemented with LIF (10 ng/µL, 300-05, Peprotech, Cranbury, NJ, USA). When ES culture became confluent in 2 days, cells were washed with HBSS (H6648, Sigma-Aldrich, Saint Louis, MO, USA), trypsinized with 0.25% trypsin-EDTA (25200-056, Sigma-Aldrich, Saint Louis, MO, USA), and 100,000 cells were passaged to a new well with inactivated MEFs.

### 2.2. Organoid Production

Retinal organoids were produced as previously described [25]. Briefly, stem cells were cultured in at least two passages on MEFs and two passages in gelatine-coated 6-well plates (CLS3516, Corning, New York, NY, USA) before the start of differentiation. On the day of the experiment, pluripotent stem cells were trypsinized (25200-056, Gibco, Thermo Fisher Scientific, Waltham, MA, USA) and aggregated in an optic vesicle induction medium (Table 4) in the ultra-low attachment 96-well plate (174925, Thermo Fisher Scientific, Waltham, MA, USA) at 3000 cells in 100 µL per well. The next day, Matrigel with reduced growth factors (354230, Corning, New York, NY, USA) was added (final concentration 2%). On day 7, the medium in wells with retinal organoids was changed to an optic-cup induction medium (Table 5). On day 9, the medium was changed for optic-cup maturation medium 1 (Table 6) and, on day 12, for optic-cup maturation medium 2 (Table 7). Medium was renewed every other day. Notably, starting from day 12, organoids were placed in an incubator with a hyperoxic atmosphere (40%) to support the survival of cells in the inner part of the aggregate.

### 2.3. Immunohistochemistry

Organoids were fixed in 4% PFA for 30 min and successively soaked in 10%, 20%, and 30% sucrose over the next three days. Organoids were then mounted in Yazulla mix (30% egg albumin and 3% gelatine in purified deionized water), poured into a mold, fast-frozen by dipping the mold in dry-ice cold isopropanol, and stored at −20 °C until use. Additionally, 12 μM sections were sliced using a cryostat (Cryostar NC80, Thermo Fisher Scientific, Waltham, MA, USA) and stored at −20 °C. Before staining, we rehydrated the sections with PBS and incubated them in a blocking solution for 1 h (goat, rabbit, or sheep serum, 0.1 Triton X-100, 1% BSA). We applied overnight the primary antibodies in the blocking solution in various concentrations (anti-Pax6 (DHSB, mouse, 1:250), anti-Rax (ab86210, rabbit, 1:200, Abcam, Cambridge, UK), anti-Vsx2 (X1180P, sheep, 1:200, Exalpha, Shirley, NY, USA), anti-Brn3a (MAB1585, mouse, 1:200, Merck, Darmstadt, Germany), anti-Gfap (Z0334, rabbit, 1:500, Dako, Glostrup, Denmark), anti-Otx2 (ab21990, rabbit, 1:200, Abcam, Cambridge, UK), anti-calbindin (CB38a, rabbit, 1:5000, Swant, Burgdorf, Switzerland), and anti-mCherry (GTX128508, rabbit, 1:250, GeneTex, Irvine, CA, USA). Secondary antibodies with a fluorescent dye (Alexa Fluor 350/488/633, Thermo Fisher Scientific, Waltham, MA, USA) were applied for 2 h, and nuclei were visualized with 4′,6-diamidino-2-phenylindole (DAPI) when possible. We used a fluorescent microscope Leica DM6 B and a confocal microscope Leica Stellaris 8 (Leica, Wetzlar, Germany) with the software LAS X 4.8.0.28989 to visualize, process, and document stainings.

### 2.4. Rax Gene Editing Preparation

**Figure 1:**
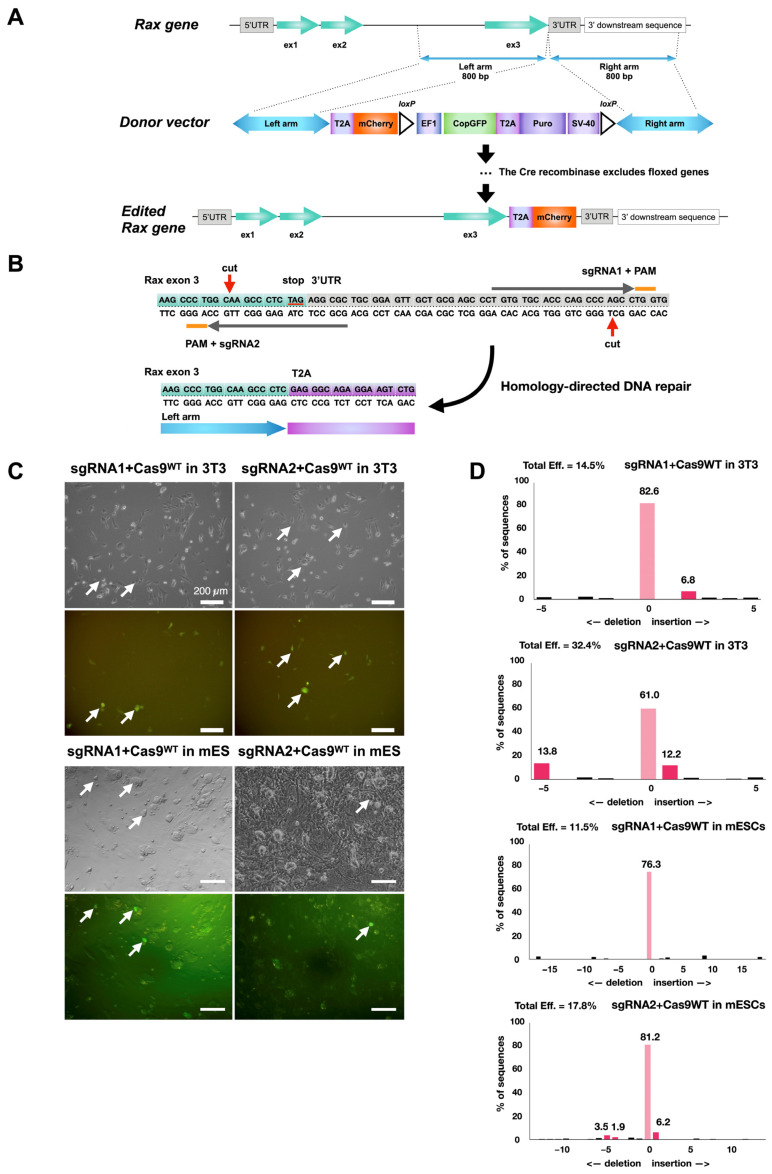
Strategy to trace *Rax*-expressing retinal progenitor cells with *Rax-mCherry* mouse reporter embryonic stem cell line. (**A**) Scheme for CRISPR-Cas9-mediated knock-in of *mCherry* transgene in mouse ES *Crx-GFP* cell line at the 3′-UTR of the *Rax* gene. (**B**) Location of nicking during CRISPR/Cas9 gene editing event and exon 3 outlook after gene editing (red arrows). Orange lines show each guide RNA’s protospacer adjacent motif (PAM). (**C**) Transfection of 3T3 and mouse embryonic stem cell lines with plasmids carrying sgRNAs and wild-type Cas9 protein. (**D**) TIDE analysis of genomic DNA from mouse 3T3 and mouse ES cells transfected with *Cas9WT/sgRNA* plasmids. Cas9WT endonuclease guided by *sgRNA1* or *sgRNA2* reporting cutting efficiency after transfection to mouse embryonic stem cells.

To fuse the gene of the fluorescent protein mCherry to the mouse *Rax* gene, we introduced two single-strand breaks using the double-nicking CRISPR/Cas9 system and edited it by supplying mouse embryonic stem cells with donor vectors carrying an insert surrounded with two 800-bp homology arms (Figure 1A). To produce plasmids carrying single-guide RNA and either Cas9(D10A) nickase or wild-type Cas9 nuclease, we used plasmids from Addgene as backbones (PX458 or PX461). Five pairs of sgRNA were designed by the CHOPCHOP web tool (http://chopchop.cbu.uib.no), and one pair was selected (Table 8, pair ranked 105 was selected). To construct the donor vector, we used the plasmid pDonor-D01 (GeneCopoeia, Rockville, MA, USA) vector as a backbone. Two synthesized *loxP*-coding sequences with few restriction enzyme sites were introduced upstream and downstream of the *copGFP-Puro* selection cassette. The DNA fragment bearing the left homology arm linked to mCherry via T2A was synthesized (GenScript, Piscataway, NJ, USA) and cloned upstream to the cassette and the 5′ *loxP* site. The right homology arm was PCR-amplified with high-fidelity DNA polymerase Phusion (New England Biolabs, Ipswich, MA, USA) and inserted downstream to the cassette and the 3′ *loxP* site. The left homology arm starts at the nucleotide 3284 in the intron 2–3 of the *Rax* gene (NC_000084.6:c65939089-65934639, GRCm38.p4) and ends right before the stop codon of the exon 3 of the Rax gene (nucleotide 4083). The right homology arm starts at the stop codon (nucleotide 4084) and extends beyond the Rax 3′ untranslated region (3′ UTR) (nucleotide 4876). All insertions were verified with Sanger sequencing (Microsynth, Balgach, Switzerland). The plasmid with the antiapoptotic factor BCL-XL was a gift from J. C. Martinou. All plasmids were prepared for electroporation experiments using the Endo-Free Plasmid Maxi Kit (12362, Qiagen, Hilden, Germany).

### 2.5. Production of Crx-GFP;Rax-mCherry Line

We used the 4D-Nucleofector (Lonza, Basel, Switzerland) system to electroporate mouse embryonic stem cells. One hundred thousand ESCs of line *Crx-GFP*, clone 6, were transfected with 0.4 µg of *sgRNA1-Cas9^D10A^* plasmid, 0.4 µg of *sgRNA2-Cas9^D10A^* plasmid, 0.8 µg of donor vector plasmid, and either 0.8 µg or 0.4 µg of BCL-XL-carrying antiapoptotic plasmid, using the program CG-104. All surviving cells were placed in one well of a 6-well plate with a feeder layer for 48 h. After expansion, we divided the cells into three separate cultures and performed puromycin selection with three concentrations in each group (0.35 µg/mL, 0.7 µg/mL, and 1.4 µg/mL) during the next 5 days and with the concentration of 1.4 µg/mL for the following 6 days in all groups. The cells selected with antibiotic concentrations of 0.35 µg/mL and 1.4 µg/mL showed the highest number of GFP-positive colonies and the best survival rate after first sub-passage to a 24-well plate. These cells were multiplied and distributed to a 96-well plate for single clone propagation in several concentrations (5, 10, 50, 150, 300, and 500 cells/well). Five single-cell-derived GFP-positive colonies from wells with a concentration of 5 and 10 cells/well were picked, expanded, and analyzed. One clone showed the correct insertion of the donor vector cargo. The floxed *copGFP-Puro* selection cassette from the *Crx-GFP;Rax-mCherry* cell line clone 1–2 was removed by transfection with Cre recombinase-containing gesicles (631449, TaKaRa, Kusatsu, Japan). GFP-negative cells were sorted at the Flow Cytometry Facility (FCF) of the University of Lausanne (UNIL) with FACS sorter Beckman Coulter MoFlo Astrios EQ (Beckman Coulter, Brea, CA, USA) and seeded into 3 96-well plates for single cell propagation. In total, 128 GFP^−^ colonies were observed. Twenty-four colonies were further expanded, and fifteen colonies were analyzed with PCR. Fourteen colonies contained mCherry and lacked a selection cassette. All 14 colonies were tested for differentiation into retinal organoids, and only 3 of them could produce organoids. The clone with the highest eye field induction efficiency (78%, cl.7) was chosen for further studies.

### 2.6. Isolation and Seeding of mCherry-Positive Cells

Day 4 stem cell-derived aggregates were dissociated, according to [26], when *Rax-mCherry* expression was detected. Cells were transferred to FCF of UNIL in transportation medium (10% KnockOut Serum (10828-028, Gibco, Thermo Fisher Scientific, Waltham, MA, USA), 1 mM EDTA (15575020, Thermo Fisher Scientific, Waltham, MA, USA), 25 mM HEPES in PBS (H0887, Sigma-Aldrich, Saint Louis, MO, USA)) on ice and sorted with BD FACSAria II (Becton, Dickinson and Company, Franklin Lakes, NJ, USA). Target cells were contrasted with mCherry-negative cells from dissociated Crx-GFP cell line-derived embryoid bodies. Additional gatings excluded cell doublets, GFP-positive cells, and dead cells (DAPI staining). Sorted mCherry-positive cells were preserved in a transportation medium on ice and delivered to the lab. Cells were pelleted by centrifugation, snap-frozen, and stored at −80 °C.

Part of the isolated cells was seeded immediately in the sorting facility in an optic vesicle induction medium addition of 20 ng/mL of EGF and FGF2 growth factors (AF-100-15, and 100-18B-50UG, both Peprotech, Cranbury, NJ, USA) and cultured in the incubator under 5% CO_2_ and at 37 °C.

### 2.7. RNA Sequencing and Analysis

RNA from isolated early retinal progenitors (mCherry-positive) derived from retinal organoids, embryonic stem cells at the origin of these progenitors, and photoreceptor cells from mice (FVB-WT from Charles River Laboratories, Wilmington, NC, USA) (*n* = 3 for mCherry-positive and mES cells, *n* = 5 for photoreceptors) were isolated with a Macherey-Nagel NucleoSpin RNA/Protein kit (740933.50, Macherey-Nagel, Düren, Germany) and sequenced. Library construction and sequencing procedures were performed by Novogene company (Beijing, China) with 150 bp paired-end reads on these samples. Fastq files of retinal progenitor cells (GFP-positive) from embryonic day 14 (E14, *n* = 2) and postnatal day 2 (P2, *n* = 2) stages were downloaded from Gene Expression Omnibus (GSE99818) [27]. The quality of the sequencing data has been evaluated with fastqc (https://www.bioinformatics.babraham.ac.uk/projects/fastqc/ last accessed on 21 September 2022).

Raw sequencing reads were mapped on the Mus musculus genome assembly GRCm38 (mm10) using Salmon (version 1.5.1) to acquire transcript abundance counts (in transcripts per million). Qualimap (version 2.2.1) was used to generate alignment data statistics and count the total number of mapped reads. In R (version 4.2.2), the resulting counts were summarized to the gene level using tximport (version 1.20.0). Genes with total counts of more than 10 were retained for differential gene expression analysis. The apeglm method in DESeq2 (version 1.32.0) was used to obtain the log fold change shrinkage. Differential gene expression analysis was performed for every pairwise comparison. The Benjamin–Hochberg procedure was applied to adjust *p*-values for statistical significance assessments. Genes were deemed differentially expressed at an absolute log2 fold change ≥ 5 and an FDR *p*-value < 0.05. MultiQC (version 1.13) was used to assess the overall quality of the entire data through each step of the protocol. All tools were employed using default parameters unless where otherwise specified.

Principal component analysis (PCA) [28] was applied to datasets by placing RNA-seq points on PC1-PC2 planes. The inferred developmental trajectory was drawn from mESCs to photoreceptors through E14 and P2 RPCs.

We used gene set enrichment analysis software (GSEA) (version 4.3.2) to perform enrichment analysis for every pairwise comparison. As input, we utilized the Gene Ontology (GO) gene set collection (version 2023.1) from the Mouse Molecular Signatures Database (MSigDB) and the output of the differential gene expression analysis (log2 transformed). Following the GSEA guidelines, we used default parameters, with the ranking metric set to ‘Diff_of_Classes’.

Volcano plots, heatmaps, PCA, and dot plots of data from expression and gene set enrichment analysis were created and performed using ggplot2 (version 3.4.2) in R (version 2.2.1).

### 2.8. Proteomics Analysis: Digestion of Protein Samples

We used three samples of mCherry-positive retinal organoid-derived cells for protein isolation containing 217,000, 385,000, and 401,000 isolated cells. To extract protein content, the Macherey-Nagel NucleoSpin RNA/Protein kit (740933.50, Macherey-Nagel, Düren, Germany) was used up to step 10. After washing with cold 60% ethanol, all samples were processed using a modified iST method [29]. Briefly, frozen cell pellets were mixed with 30 µL miST lysis buffer (1% Sodium deoxycholate, 100 mM Tris pH 8.6, 10 mM DTT). Resuspended samples were warmed up for 5 min at 95 °C. Alkylation of reduced disulfides was achieved by adding ¼ volume (25 µL) of 160 mM chloroacetamide (final 32 mM) and incubating for 45 min at 25 °C in the darkness. Samples were modified to 3 mM EDTA and digested with 0.2 µg Trypsin/Lys-C mix (Promega, Madison, WI, USA) for 60 min at 37 °C. Next, a second 1 h digestion with an additional Trypsin/Lys-C aliquot followed. Two sample volumes of isopropanol mixed with 1% TFA were added to remove sodium deoxycholate. The centrifugation was used to desalt the samples on a strong cation exchange (SCX) plate (Oasis MCX; Waters Corp., Milford, CT, USA). Peptides were eluted in 250 µL of 80% MeCN, 19% water, and 1% (*v*/*v*) ammonia after washing with isopropanol/1%TFA. Eluates were dried and resuspended in 200 µL of 2% MeCN and 0.1% TFA after SCX desalting.

### 2.9. Liquid Chromatography–Tandem Mass Spectrometry (LC-MS/MS)

LC-MS/MS analysis was performed on a TIMS-TOF Pro (Bruker, Bremen, Germany) mass spectrometer interconnected through a nanospray ion source (“captive spray”) to an Ultimate 3000 RSLCnano HPLC system (Dionex, Sunnyvale, CA, USA). Peptides were split on a reversed-phase custom-packed 40 cm C18 column (75 μM ID, 100Å, Reprosil Pur 1.9 µM particles, Dr. Maisch, Ammerbuch, Germany) at a flow rate of 0.250 µL/min with a 6–27% acetonitrile gradient over 92 min, followed by a ramp to 45% over 15 min and to 95% over 5 min (all solvents contained 0.1% formic acid). Data-dependent acquisition (DDA) was conducted using a standard TIMS PASEF method [30], with ion accumulation lasting 100 ms for every MS1 survey scan and the TIMS-coupled MS2 scans. The duty cycle was fixed at 100%. For each TIMS scan, up to ten precursors were targeted. Precursor isolation was made with a 2 Th window below or a 3 Th window above *m*/*z* = 800. The minimum threshold intensity to select the precursor was set at 2500. Next, the precursors were targeted more than once to attain a minimum target total intensity of 20,000 if the inclusion list permitted. The collision energy was increased linearly based exclusively on the 1/k_0_ values from 20 (at 1/k_0_ = 0.6) to 59 eV (at 1/k_0_ = 1.6). The total duration of a scanning cycle, which comprised one survey and 10 MS2 TIMS scans, was 1.16 s. Precursors were targeted again in the following cycles in case of signal rise by a factor of not less than 4. If the precursors were selected in one cycle, they were banned for further selection for 1 min. The mass resolution in all MS measurements was around 35,000.

### 2.10. Analysis of LC-MS/MS Data

Tandem MS data were analyzed with the MaxQuant program (version 1.6.14.0) [31], which incorporated the Andromeda search engine [32]. The UniProt reference proteome (RefProt) database for *Mus musculus* (version of 4 June 2021) and sequences of widespread contaminants were used. Trypsin, which cleaves at lysine and arginine residues, was set as an acting enzyme, and two missed cleavages were authorized. Carbamidomethylation of cysteine was set as a constant modification. Peptide N-terminal acetylation and methionine oxidation were specified as changing modifications. Filtering at 1% FDR at both the peptide and protein levels with default MaxQuant parameters was used for all findings. MaxQuant data were later analyzed with the Perseus program [33]. LFQ values [34] were log2 transformed and used for quantitation afterward.

### 2.11. Embryoid Body Differentiation Assay

The pluripotency of the generated Crx-GFP;Rax-mCherry cell line was investigated with an embryoid body (EB) differentiation assay according to [35] with some changes. The cells were cultured as usual, and on day 0, they were trypsinized and placed into a bacterial culture-grade Petri dish (430589, Corning, New York, NY, USA) without LIF for 3 days. Embryoid bodies spontaneously formed and were plated onto Matrigel-coated tissue-culture-treated 12-well plates (Costar 3513, Corning, New York, NY, USA), where they attached for 3–6 days. EBs were fixed with 4% PFA for a quarter of an hour, cleaned with PBS, and immunolabeled against anti-alpha-fetoprotein (endoderm marker, MAB1368, Merck, Darmstadt, Germany), anti-smooth muscle actin (mesoderm marker, M0851, clone 1A4, Dako, Glostrup, Denmark) and anti-beta-tubulin3/TUJ1 (ectoderm marker, GTX631836, GeneTex, Irvine, CA, USA).

## 3. Results

### 3.1. Knock-In of mCherry Sequence After the Rax Gene

In order to produce a reporter mouse pluripotent stem cell line that would allow us to identify and trace retinal progenitors (RPCs), an mCherry sequence was inserted after the *Rax* gene. *Rax* is one of the eye field transcription factors coordinating optic vesicle formation and, later, eye development [36,37]. At first, it is expressed in the eye field cells and then in retinal progenitors, and, later, it appears at a low level in rods after neurogenesis. The insertion was performed in the mouse embryonic stem (ES) Crx-GFP cell line [23].

Among all available gene editing strategies, the CRISPR/Cas9 technology is considered to be the most efficient and reliable [38]. However, given the increased risk of off-target mutations with wild-type Cas9, the approach selected was to use Cas9^D10A^ nickase [39]. In this method, two Cas9n-mediated nicks on opposite DNA strands form double-stranded breaks with sticky ends, and subsequent DNA repair occurs according to the desired editing. *mCherry* was selected as a reporter gene for its small DNA sequence size and its high fluorescence properties [40]. The *mCherry* sequence was introduced into a *copGFP-Puro* selection cassette plasmid to be inserted after the last exon of the *Rax* gene by gene editing (Figure 1A). A web tool, CHOPCHOP (http://chopchop.cbu.uib.no), was used to design single-guide RNA (*sgRNA*) sequences for the nicking of the desired target [41]. Five proposed *sgRNA* pairs (Table 8) were screened according to criteria described in the literature—offset *sgRNA* spacing between 10 and 31 bp, 5′ overhang at the double-strand break, and minimal disruption of the protein-coding region by the enzyme [39,42].

After this analysis, *sgRNA* pair 105 (Table 8) was selected for further use (Figure 1B). The ability of the selected *sgRNAs* to target the correct region was measured after cloning into plasmids with wild-type *Cas9*, transfection in 3T3 cells (Figure 1C), and TIDE analysis [43] (Figure 1D). A cutting efficiency of nearly 15% and 32% for each single-guide RNA was reached, close to the 3T3 cell transfection efficiency (~20–30%), suggesting high efficiency of *sgRNAs* to engage the Cas9 protein and induce double-strand breaks. The same test was repeated with mES cells. Cutting efficiency diminished to 11% and 18%, likely due to the lower viability of stem cells upon electroporation (Figure 1C,D).

**Figure 2:**
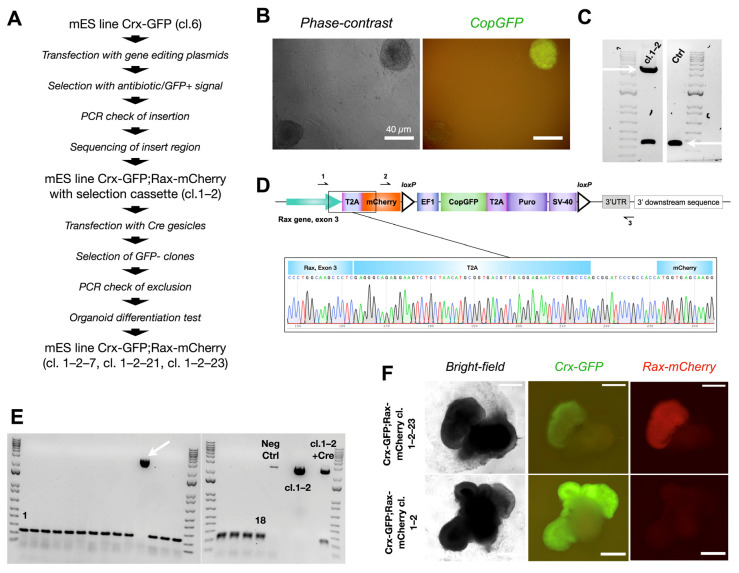
Production of mouse embryonic stem cell reporter line Crx-GFP;Rax-mCherry. (**A**) Strategy to generate reporter Crx-GFP;Rax-mCherry mouse embryonic stem cell line. (**B**) GFP-positive mouse ESC colony after cassette insertion. (**C**) Confirmed monoallelic insertion (upper arrow, primers 1–3 from Figure 2D, 3659 bp) of the mCherry-CopGFP-Puro construct into mouse ESC clone 1–2 by PCR. Both lower bands represent the end of exon 3 of the Rax gene without modification (lower arrow, primers 1–3 from Figure 2D, 355 bp). (**D**) Scheme of exon 3 of the Rax gene after the insertion and a chromatogram of the “Rax gene-T2A-mCherry” region. (**E**) PCR test of 18 clones to confirm exclusion of selection cassette CopGFP-Puro after Cre expression. One clone has an insertion (arrow). All PCR products were amplified with primers 2–3 from Figure 2D, with resultant bands of 194 bp or 2742 bp. (**F**) Differentiation test. Three clones of Crx-GFP;Rax-mCherry mES cell line (cl.1–2–7, cl. 1–2–21, cl. 1–2–23) could produce retinal organoids with a clear expression of mCherry.

A previously generated mES cell line, *Crx-GFP* [23], was used for gene editing. Cells were transfected with nick-forming plasmids (Cas9^D10A^+sgRNAs), a plasmid carrying the donor sequence, and a plasmid encoding the antiapoptotic factor BCL-XL (Figure 2A). The cells were then exposed to puromycin to expand antibiotic-resistant clones. The *copGFP* expression of selected clones was verified by microscopy (Figure 2B). Single-cell clones were propagated and cultured to perform PCR analysis and DNA sequencing. The analysis confirmed an errorless heterozygous insertion of the *copGFP-Puro* construct into one clone (cl.1–2, Figure 2C,D). The cells were transfected with Cre-recombinase-carrying vesicles (TaKaRa) to delete the floxed selection cassette containing *copGFP* and puromycin-resistance genes. Single-cell clones were grown over 4–5 passages, GFP-negative ones were selected, and the excision of the selection cassette was verified by PCR. 

**Figure 3:**
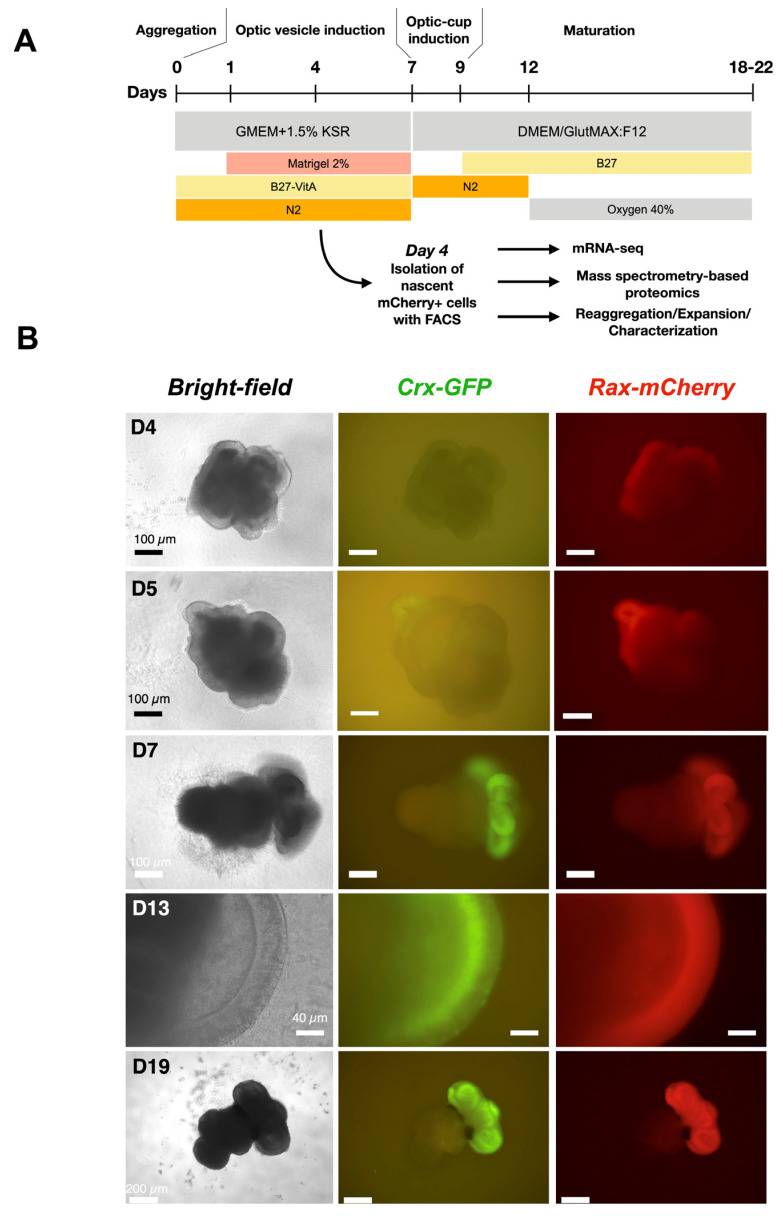
Development of retinal organoids derived from cl.1–2–7 of *Crx-GFP;Rax-mCherry* reporter mouse stem cell line. (**A**) Protocol for mouse retinal organoid differentiation [23]. (**B**) Retinal organoids at different time points of differentiation. Retinal tissue sprouts from the mother aggregate.

Out of 18 clones, 17 lacked the *copGFP-Puro* construct (Figure 2E). Cells from the 17 clones were differentiated according to our published protocol [25] (details in the Materials and Methods part and scheme in Figure 3A). Surprisingly, only cells from three clones differentiated into organoids with an optic-cup organization with Crx-GFP positive cells (clones 1–2–7, 1–2–21, and 1–2–23, Figure 2F). The embryoid body differentiation assay was performed with cells of cl. 1–2–7 and confirmed the preservation of pluripotency (Supplementary Figure S3). Interestingly, unlike in the organoids derived from the subsequent copGFP-Puro-negative clones, no mCherry fluorescence was observed in the retinal organoids derived from the parental cl. 1–2, suggesting that the selection cassette precluded the formation of a functional fluorescent protein (Figure 2F).

### 3.2. mCherry Is Expressed During Early Retinal Organoid Development

Among the three clones, clone 1–2–7 contained the organoids with the highest optic-cup induction efficiency (78%) and was used for further experiments. In differentiation experiments, the expression of *mCherry* started on day 4 after aggregation (Figure 3B), suggesting the place of eye field induction, whereas the expression of GFP from CRX-positive cells, although expected at this developmental stage, was not visible. The next day, the expression of *Crx* began. In the following days, the organoid matured, and mCherry remained in the whole width of the retina, showing that retinal progenitors are still highly present, while GFP was restricted to photoreceptor precursors and immature photoreceptors aligned at the basal surface of the retina (Figure 3B, day 13). At a later stage, on day 19 (Figure 3B), both fluorescent signals largely overlapped (Supplementary Figure S2), indicating the end of neurogenesis. *Rax* expression in rods was thus confirmed [44]. In conclusion, we have generated a new cell line that allows the tracking of probable retinal progenitors (RAX-positive cells) and photoreceptor precursors (CRX-positive cells) during retinogenesis.

### 3.3. Cell Type Composition of the Crx-GFP;Rax-mCherry Retinal Organoids

**Figure 4:**
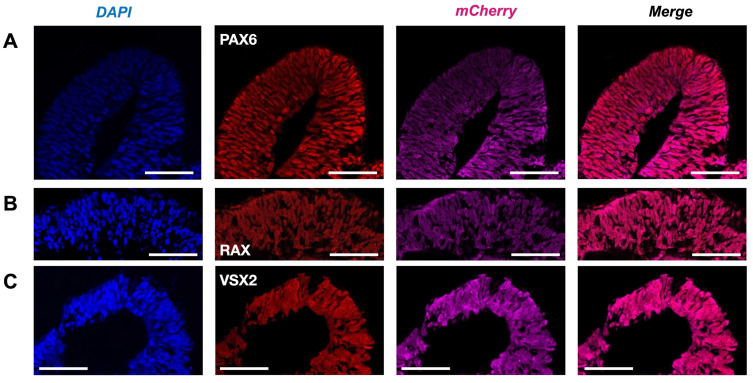
Retinal progenitors in Crx-GFP;Rax-mCherry-derived retinal organoids. (**A**) Anti-VSX2 labeling at day 9 (D9). (**B**) Anti-RAX labeling at day 9 (D9). (**C**) Anti-PAX6 labeling at day 9 (D9). Scale bar—50 µm.

**Figure 5:**
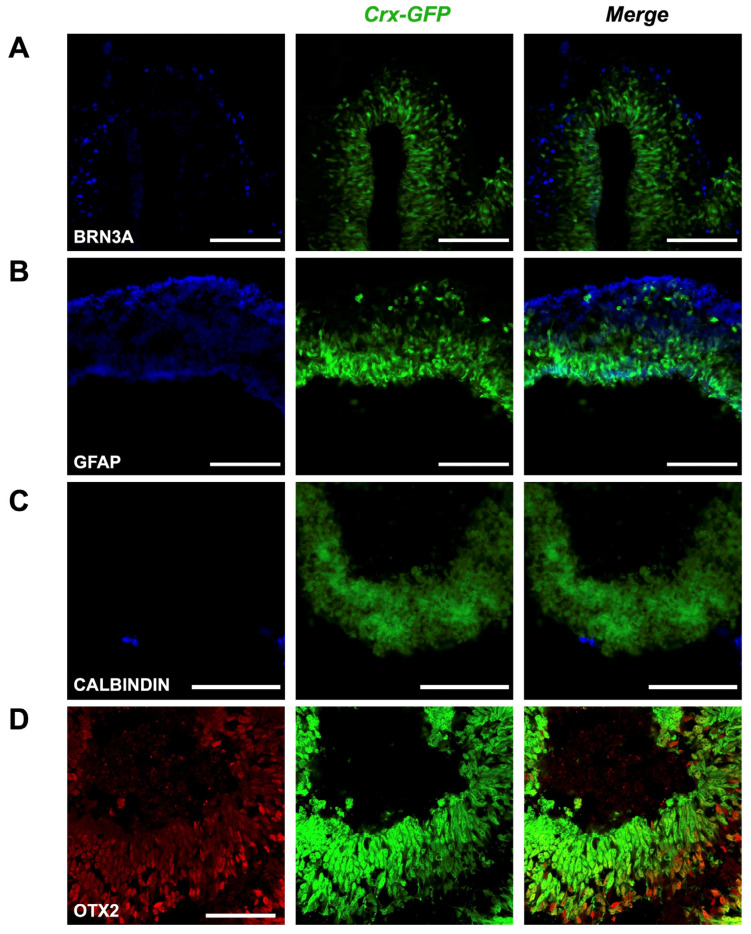
Presence of various cell types in mature retinal organoids. (**A**) BRN3A is a marker of retinal ganglion cells. (**B**) GFAP is a marker of Müller glial cells. (**C**) CALBINDIN is a marker of horizontal and amacrine cells. (**D**) OTX2 is a marker of bipolar cells. Scale bar—100 µm for (**A**–**C**), 50 µm for (**D**).

The presence of the main retinal cell types produced after the insertion of the *mCherry* transgene was validated to ascertain that no perturbation had occurred during the development of the *Crx-GFP;Rax-mCherry* retinal organoids (Figure 4 and Figure 5). The fully differentiated mouse retina comprises six neuronal and one glial cell type, which appear from retinal progenitor cells during retinogenesis. Retinal progenitors are differentiated from eye-field neuroepithelial cells that express eye-field transcription factors (EFTFs, i.e., PAX6, OTX2, SOX2, LHX2, RAX, SIX3, SIX6, and NR2E1). The differentiation of the seven cell types occurs during a well-organized three-stage spatiotemporal patterning. Retinal ganglion cells, GABAergic amacrine cells, horizontal cells, and cone photoreceptor cells appear from early retinal progenitors during the first stage. The second stage gives rise to other types of amacrine cells and rods. Finally, bipolar and Müller glial cells are differentiated from late progenitors [45]. The mouse retina is formed within two weeks, from around E10 to P5, and matures until P30 [45,46]. To validate the correct differentiation of our model, specific cell markers were revealed by immunohistochemical labeling at different time points of the organoid development. Since our retinal organoids started to express RAX at days 4–5, we set day 4 as the starting point of retinogenesis. First, we aimed to characterize Rax-mCherry-positive cells as potential retinal progenitors at a later stage of development (day 9). RAX and PAX6 transcription factors have been described to first appear in eye field neuroepithelial progenitor cells and later remain in retinal progenitors. In the mature retina, RAX is present in rods and Müller glial cells, and PAX6 is expressed in retinal ganglion cells (RGCs) and some amacrine cells [47]. Transcription factor VSX2 is considered to be the first retinal progenitor marker after eye field induction. After retinal progenitor specification, it ensures its proliferation and maintenance while suppressing RPE-promoting transcription factor MITF. Immuno-labeling against retinal progenitor markers (RAX, PAX6, and VSX2) was performed to determine the degree of lineage commitment of the mCherry-positive cells. The markers were visualized with red secondary antibodies to reveal potential coexpression with Rax-mCherry-positive cells (in magenta). As a result, the nuclear-localized signals of anti-RAX, anti-PAX6, and anti-VSX2 overlapped with mCherry-positive cells in retinal organoids at day 9 of differentiation and supported the progenitor identity (Figure 4A–C).

Next, we expected to observe the formation of all main cell types by days 17–18, assuming the developmental rate in vitro to be similar to the in vivo situation. We therefore collected organoids aged between 17 and 25 days to label different retinal cell types. Staining of BRN3A (blue) marked a dispersed population of retinal ganglion cells of the evaginated optic vesicle (Figure 5A); reactivity to GFAP (blue) in the same region labeled an outer foot of Müller glia cells (Figure 5B); scant potential amacrine and horizontal cells appeared with anti-Calbindin staining (blue, Figure 5C), and sparse bipolar cells were visualized with OTX2 labeling (red, Figure 5D). The green labeling corresponds to the GFP expression controlled by *Crx* inherent to cone and rod photoreceptor cells. These results show that our gene editing approach did not alter the potency of pluripotent stem cells to generate retinal organoids containing the different retinal cell types. The activity of retinal progenitor cells at different retinal developmental stages is also revealed, as well as their ability to proliferate (extension of the mCherry-positive cell population during early time points) and differentiate into all main retinal types.

### 3.4. Rax-mCherry-Positive Cells Have Characteristics of Retinal Progenitors

#### 3.4.1. Isolated Cells Proliferate in the Presence of EGF and FGF2

**Figure 6:**
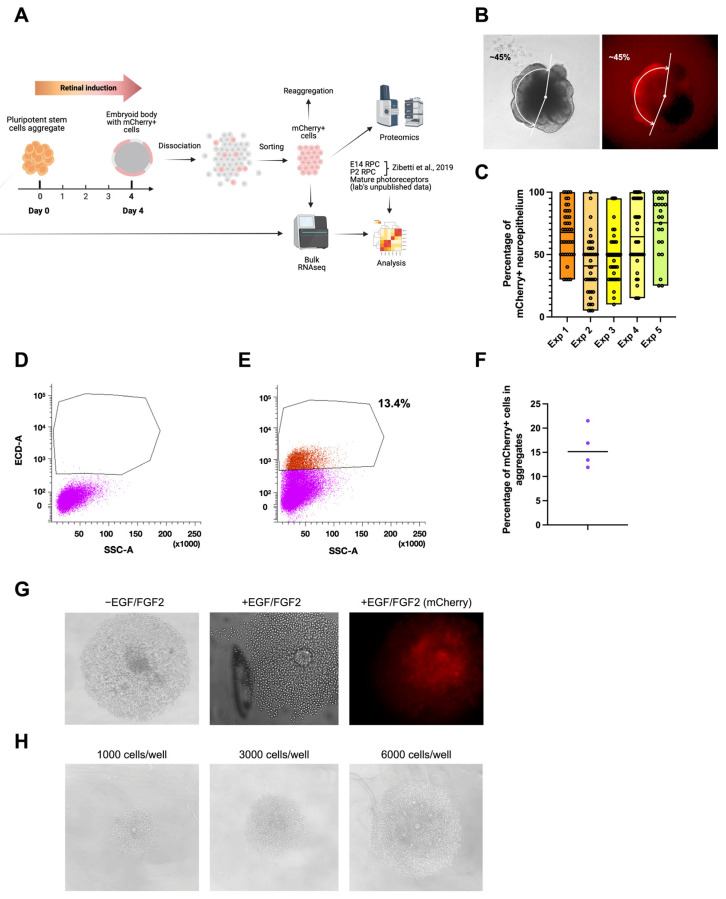
Isolation and analysis of mCherry-positive cells from day 4 mouse retinal organoids. (**A**) Scheme of the mCherry-positive cell isolation and analysis. (**B**) Estimation of mCherry+ part of neuroepithelium in day 4 (D4) embryoid body. (**C**) Part of mCherry+ neuroepithelium in *Crx-GFP;Rax-mCherry* stem cell line-derived aggregates in 5 experiments (Exp. 1, *n* = 40; Exp. 2, *n* = 40; Exp. 3, *n* = 37; Exp. 4, *n* = 40, Exp. 5, *n* = 26). (**D**) Negative control cells from *Crx-GFP*-derived stem cell aggregates. (**E**) The population of sorted mCherry+ cells from dissociated *Crx-GFP;Rax-mCherry* stem cell line-derived aggregates. (**F**) Part of mCherry+ cells in FACS-analyzed aggregates in 4 experiments. (**G**) Aggregation of mCherry+ cells with and without growth factors EGF and FGF2 in a 96-well plate with a U-shape bottom and low-adhesion surface. (**H**) Aggregation of mCherry+ cells with different cell densities [27].

The mCherry signal appeared in developing embryoid bodies on the fourth day after the aggregation of stem cells and induction of retinal fate. We hypothesized that mCherry-positive cells at this stage are early retinal progenitors. To test this hypothesis, we used flow cytometry to isolate mCherry-expressing cells on day 4 to assess their identity and proliferative potential (Figure 6A). Before cell isolation, we estimated the proportion of mCherry-positive neuroepithelium (Figure 6B). The analyzed experiments were characterized by a high heterogeneity of RAX-positive area size but also by a 100% induction efficiency, meaning that all of the embryoid bodies had a potential retina-committed zone (Figure 6C). This finding has to be confirmed with the presence of other EFTFs. The aggregates were dissociated into single cells [26], and mCherry-positive cells were FACS-sorted. Negative control embryoid bodies from a stem cell line not expressing mCherry were produced to define the sorting gates (Figure 6D,E). The part of isolated mCherry-positive cells in four sorting experiments was between 13 and 22% (Figure 6F). Isolated cells were then seeded to a 96-well plate with a low attachment surface and a U-shape bottom in an optic vesicle induction medium at a concentration of 3000 cells/well. Since the cells did not survive in the regular medium, EGF and basic FGF (FGF2) growth factors (concentration 20 ng/mL, [48]) were added in the subsequent experiments, allowing the formation of mCherry-positive cell colonies in the next two days (Figure 6G). The increase in seeding density led to the formation of more colonies (Figure 6H). Taken together, these findings suggest that mCherry-positive cells can proliferate and carry the potential retinal progenitor identity or adopt it soon after isolation. These cells are hereinafter referred to as “D4 RPCs”.

#### 3.4.2. Transcriptomic Analysis Implies Retinal Progenitor Cell Fate Commitment of Isolated mCherry-Positive Cells

**Figure 7:**
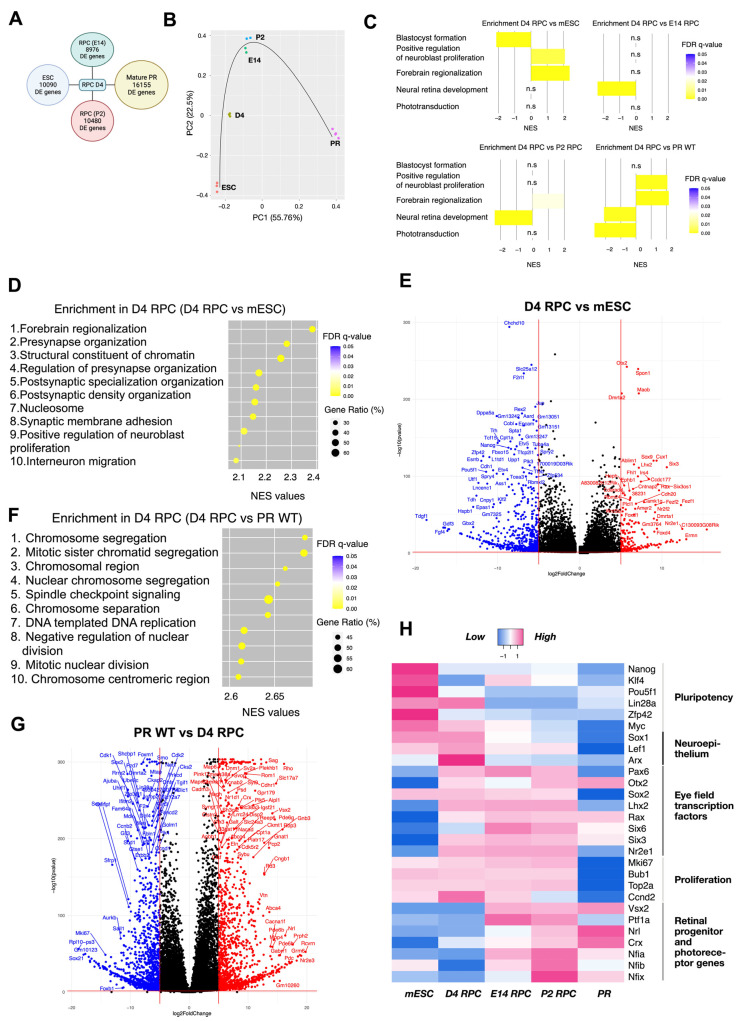
Transcriptomic analysis of day 4 mCherry-positive cells from mouse retinal organoids. (**A**) Number of differentially expressed genes with adjusted *p*-value less than 0.05 in D4 RPC compared to mESC, E14, P2 RPC, and wild-type mature PR. (**B**) Principal component analysis of mESC, D4 RPC, E14 RPC, P2 RPC, and wild-type mature PR. The black line indicates the approximate developmental trajectory. (**C**) Enrichment of 5 selected gene sets in D4 RPC compared to mESC, E14 RPC, P2 RPC, and wild-type mature PR. NES—normalized enrichment score, gene sets enriched in D4 RPC have positive NES, and gene sets enriched in another group have negative NES. (**D**) Top 10 enriched gene sets in comparison “D4 RPC versus mESC”. Gene ratio indicates the number of genes of the leading edge subset that actively contribute to the enrichment score. (**E**) Volcano plots for differentially expressed genes of Crx-GFP;Rax-mCherry D4 RPC compared to mouse embryonic stem cells. (**F**) Top 10 enriched gene sets in comparison “D4 RPC versus WT PR”. (**G**) Volcano plots for differentially expressed genes of Crx-GFP;Rax-mCherry D4 RPC compared to mouse wild-type mature PR. (**H**) Heatmap of gene expression in mESC, D4 RPC, E14 RPC, P2 RPC, and wild-type mature PR. Selected genes are grouped into five categories. mESC—mouse embryonic stem cells, RPC—retinal progenitor cells, PR—photoreceptors. n.s.: non-significant.

Deeper characterization of the isolated cells was performed by transcriptome analysis and compared to either Crx-GFP;Rax-mCherry original pluripotent stem cells, retinal progenitor cells isolated at E14 or P2 (publicly available data: [27]), or isolated mature photoreceptors (unpublished data from our lab). Differentially expressed gene (DEG) analysis showed a high number of differentially regulated genes between isolated D4 RPCs and each of the four other groups. The highest difference was seen in comparison with mature photoreceptors (PRs) (Figure 7A). Principal component analysis (PCA) positioned the D4 RPCs on the trajectory between stem cells and E14 and P2 retinal progenitor (E14 RPC/P2 RPC) populations (Figure 7B). Gene set enrichment analysis (GSEA) was conducted to understand which biological processes defined the organoid D4 RPC population compared to each group.

The normalized enriched score (NES) of the topmost enriched and biologically meaningful gene sets in comparison to D4 RPCs are as follows: “Blastocyst formation” (NES = 2.14, FDR = 0.0001): third top enriched gene set in mESC versus D4 RPC; “Forebrain regionalization” (NES = 2.38, FDR = 0.0001): first top enriched gene set in D4 RPC versus mESC; “Positive regulation of neuroblast proliferation” (NES = 2.09, FDR = 0.003): ninth top enriched gene set in D4 RPC versus mESC; “Neural retina development” (NES = 2.37, FDR = 0.0001 and NES = 2.35, FDR = 0.0001): first top enriched gene set in both E14 and P2 versus D4 RPC; and “Phototransduction” (NES = 2.73, FDR = 0.0001): eighth enriched gene set in PR WT versus D4 RPC. These genesets highlight the position of D4 RPCs relative to the four other groups.

These characteristic gene sets reflect the differentiation process from pluripotent stem cells to retinal progenitors and, finally, to retinal cell types, such as photoreceptors. Indeed, it demonstrated the differentiation of D4 RPC from mESC towards a neuronal lineage/eye primordia (“Positive regulation of neuroblast proliferation” and “Forebrain regionalization”) but low specification to E14 and P2 RPC (“Neural retina development”) and photoreceptive immaturity (“Phototransduction”) (Figure 7C).

More precisely, juxtaposed with mES cells, D4 RPCs show enrichment of gene sets related to brain neurogenesis (synapse formation, brain regionalization, proliferation, and migration of neurons) and chromatin changes in D4 RPCs (Figure 7D). In detail, among the 11 topmost enriched genes of the “Forebrain regionalization” gene set, 4 are EFTFs (*Six3*, *Lhx2*, *Otx2*, and *Pax6*), and 3 are indirectly involved in retinal development since they were significantly downregulated in Rax-knockout retinal organoids (*Fezf1/Fezf2* and *Wnt7b*, [49]; *Nr2f1*, [50]). The “Positive regulation of neuroblast proliferation” gene set additionally features, first, the EFTF *Nr2e1* together with *Pax6*; second, the transcription repression factor *Foxg1* involved in telencephalon and retinal development; and third, members of WNT and SHH signaling (*Frizzled*, *Smo*, and *Gli3*), crucial for eye field formation and RPC proliferation [45,51,52]. Conversely, gene sets linked to stem cell maintenance (for example, “Blastocyst formation” and “Blastocyst development”) were enriched in stem cells, i.e., downregulated in D4 RPC compared to mESC (Figure 7C and Figure S1). The comparison of deregulated genes in D4 RPC with stem cells, visible in a volcano plot (Figure 7E), highlighted the downregulation in D4 RPC of major regulators of pluripotency (e.g., *Pou5f1, Zfp42, Nanog*). In contrast, EFTFs (*Otx2*, *Lhx2*, *Six3*, *Rax*, and *Nr2e1*) were among the most upregulated.

On the other hand, the comparison of D4 RPCs with photoreceptors indicated the enrichment of pathways linked to light detection in photoreceptors (Figure 7C,G and Figure S1) and proliferation-related processes in D4 RPCs, with 17 out of the top 20 most enriched gene sets in D4 RPCs related to cell division (Figure 7G). The 20 topmost enriched genes in D4 RPCs included many proliferation markers, such as *Mki67*, *Ccnd2* (cyclin D2), *Hmga2*, *Top2a* (DNA topoisomerase II alpha), *Bub1*, and *Kif15* (Supplementary Table S1, Figure 7H). Similarly, 13 out of the top 20 enriched gene sets in photoreceptors compared to D4 RPCs describe their structure or function (Figure 7C,G and Figure S1), including characteristic genes such as neural retina leucine zipper (*Nrl*), rhodopsin (*Rho*), retinol-binding protein 3 (*Rbp3*), recoverin (*Rcvrn*), and peripherin (*Prph2*) (Supplementary Table S2, Figure 7F).

A comparison of D4 RPCs with E14 and P2 RPCs shows close trends with enrichment of gene sets related to retinal development in E14 and P2 RPCs (7 and 5 gene sets, respectively, out of the top 10 enriched are retina-related). The top enriched “Neural retina development” gene set (Figure 7C) presents the earliest marker of retinal progenitor cells *Vsx2* as the most enriched gene. In contrast, the “Midbrain development” gene set was among the most enriched biologically relevant gene sets in D4 RPCs compared to E14 and P2 RPCs. This gene set includes important modulators of embryonic neurogenesis, such as *Foxb1*, *Hes3*, *Otx1*, *Dkk1*, *Wnt1*, and 3 FGF receptors (*Fgfr1*, *Fgfr2*, and *Fgfr3*).

Next, we focused on the expression of selected genes, characteristic of relevant biological processes and identities, as described in Figure 7H (for clarification: markers of retinal progenitors are *Vsx2*, *Nfia/b/x*, and *Ptf1a*; for mature photoreceptors, they are *Nrl* and *Crx*). These results were the first to exemplify the striking difference between D4 RPCs and pluripotent stem cells. The downregulation of pluripotency genes and the upregulation of EFTFs (except for the unaltered *Sox2* and *Lin28a* involved in eye initiation) evidenced an identity modification. Both groups were characterized by an unchanged high expression of proliferation and neuroepithelium genes and not-yet-active retinal progenitor genes. D4 RPCs were also remarkably different from photoreceptors. Genes from all groups except pluripotency were deregulated—all proliferation, neuroepithelium, and many EFTF genes were inhibited in photoreceptors (some EFTFs remained active in mature rods (*Rax*) and bipolar cells (*Otx2*, [53])), whereas retinal progenitor and photoreceptor marker genes were activated (except *Ptf1a,* which was active in mature amacrine and horizontal cells). On this heatmap, too, D4 RPCs appeared more similar to retinal progenitor cells. Retina-unrelated pluripotency genes were inactivated, and high expression of proliferation and EFTF genes was observed in all three groups. However, compared to E14 and P2 RPCs, D4 RPCs expressed a lower level of the retinal progenitor genes (notably, the first RPC marker *Vsx2* was inactive) and a higher level of neuroepithelium genes. In summary, this part of the analysis delineates the trend of isolated day 4 cells to be distinct from pluripotent stem cells and mature photoreceptors but closer to the eye field expressing neuroepithelium and retinal progenitors. These data attest that our D4 RPCs develop early RPC features.

#### 3.4.3. Protein Content Corroborates Retinal Progenitor Identity

**Figure 8:**
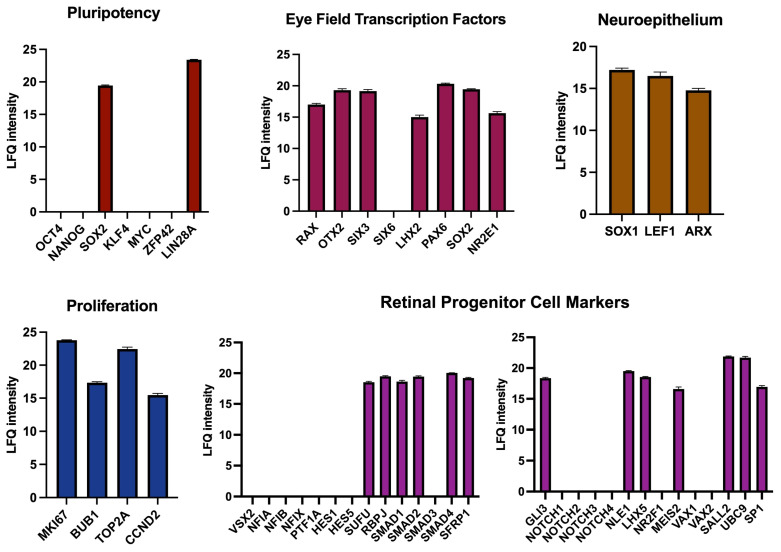
Mass spectroscopy-based proteomic analysis of D4 mCherry-positive cells from mouse retinal organoids. The protein composition of mCherry+ cells is compared to the characteristic protein content of various cell types or processes.

Finally, we aimed to support our findings from transcriptomic data with a proteomic study of D4 RPCs (Figure 8). Protein content data obtained by liquid chromatography with tandem mass spectrometry confirmed the presence of 2 out of the 7 major pluripotency regulators (SOX2 and LIN28A) as observed at the RNA level. SOX2 is necessary for neural and eye induction and is present in early progenitors [52]. The high expression of LIN28A in early progenitors was reported at a stage as early as E12 for mouse retina [54]. All EFTFs seen at the RNA level were also detected at the protein level (RAX, OTX2, SIX3, SOX2, LHX2, PAX6, and NR2E1), as well as proliferation and neuroepithelium regulators. Among the retinal progenitor genes, some critical RPC proliferation and differentiation proteins were present (RBPJ, SMAD proteins, and MEIS2), while some others, notably VSX2, NFI factors, HES1, HES5, and NOTCH receptors, were not detected. In conclusion, the protein composition of D4 RPCs confirms their early commitment to retinal progenitor cells.

## 4. Discussion

We produced a novel reporter mouse embryonic stem cell line, *Crx-GFP;Rax-mCherry*, efficient in separately tracing retinal progenitor cells and photoreceptors. We show here the possibility of following during the whole retinogenesis process the early retinal progenitors derived from day 4 embryoid bodies committed to inducing optic-cup-like formations and then more mature RPCs. To the best of our knowledge, this cell line is the only existing dual-reporter mouse embryonic stem cell line where two genes are crucial in the context of retina development. Similar human stem cell lines combine eye field transcription factor SIX6 and photoreceptor regulator CRX or retinal ganglion cell marker POU4F2 as discussed below.

### 4.1. Eye Field Transcription Factor Gene Reporter Lines in Retina Research

Stem cell lines with an eye field transcription factor gene reporter are valuable tools in retina research. The Sasai group used a mouse *Rax-GFP* line in the pioneering study of retina organoids [6]. This cell line served to detect and capture the progression of mouse optic vesicles and optic-cup formation in vitro in real time with two-photon microscopy. However, the intensity of the fluorescent signal was poor, limiting certain studies. Another group generated a human EGFP plasmid under the control of the *Rax* promoter and used it to study regulatory regions of the *Rax* gene [55], but no stable cell line was established. The dual human reporter cell line *Six6-GFP/Pouf2-tdTomato* helped to visualize developing retina and retinal ganglion cells separated from hypothalamic and midbrain tissues, aiming to optimize a protocol for retinal induction and to study transcriptomes of early retinal and non-retinal SIX6+ tissues [56]. Another known double reporter human PSC line, *SIX6-p2A-eGFP/CRX-p2A-h2b-mRuby3*, was created to produce retinal organoids and generate a detailed transcriptomic database over the span of organoid development [57]. Several human and mouse *Pax6* reporter lines were created. Anchan and colleagues used the mouse ES *Pax6-GFP* cell line to find conditions to generate lens cells in vitro [58]. Wu and co-authors created the *PAX6^WT/EGFP^* human PSC line to develop protocols generating neural progenitor cells in 2D culture [59]. Mike Karl’s group used mouse and human Pax6-GFP lines to refine a retinal organoid culture method [60]. A mouse *Otx2-GFP* line was used to visualize OTX2 protein distribution during brain development and its cellular localization in retinal cell types [61]. Chick *Otx2-GFP* cells helped to isolate retinal progenitors at the end of retinogenesis (chick retina at stage E7 corresponds to mouse retina at stage P2) and study the role of OTX2 during the specification of progenitors to photoreceptors. Also, the CRISPR/Cas9-controlled switch in the line helped to conditionally deactivate *Otx2* during neurogenesis [62]. The group of Boaz Levi used three distinct engineered reporter cell lines (*SOX2^Cit/+^, OTX2^Cit/+^*, and *PAX6^Cit/+^*) to build a transcriptome signature of cell fate decisions during early brain development. They also confirmed the essential role of Wnt/b-catenin signaling for brain region specification [63]. To conclude, we created the first mouse dual reporter cell line marking progenitor-specific and photoreceptor-specific genes simultaneously.

### 4.2. Rax Gene Reporter with Biallelic Editing

The end of the *Rax* exon-3 was successfully modified with the mCherry construct in one allele without introducing indel mutations at insertion sites. Live-imaging experiments proved a stable and robust fluorescence detection. However, immunolabeling of organoid sections showed that the mCherry proteins were dispersed in the cell cytoplasm and bleached quickly during microscopy imaging. Another concern was that the monoallelic knock-in threatened the construct’s genetic stability during mitosis. Although biallelic insertion might be more genetically stable and yield extra fluorescent proteins, additional rounds of gene editing and clone expansion would increase the age of the stem cells, as well as the risk of introducing genetic mutations, and reduce their differentiation potential [64,65]. Nonetheless, our cell line with the double markers is highly sensitive and allows us to detect RPCs very early during organoid development and to simultaneously follow the events of photoreceptor differentiation.

### 4.3. In Vitro Development in Crx-GFP;Rax-mCherry Retinal Organoids

Our in vitro model shows a distinctive feature of accelerated retinogenesis. We observed neural induction at a time point similar to in vivo progression. Starting the stem cell culture (day 0) corresponded to the embryonic time of inner cell mass formation (E3.5). During neural induction, the eye field was formed on some parts of the aggregate on day 4 (first Rax-positive cells), while in vivo, this process occurred at E7.5, also 4 days later [51]. However, the appearance of Crx-positive cells occurred in vivo at E12.5 [9], whereas the first Crx-positive cells could already be seen on day 5 in our organoids.

### 4.4. Transcriptomic Analysis

Our analysis of transcriptomics, proteomics, and proliferation tests of mCherry-positive cells isolated from day 4 mouse retinal organoids places their identity between neuroectodermal eye field cells and early retinal progenitor cells. While the lack of expression of the first retinal progenitor marker *Vsx2* and the high expression level of some pluripotent markers (*c-Myc* and *Pou5f1* (*Oct3/4*) but not *Nanog*, *Klf4*, and *Zfp42*) supports an identity closer to neuroectodermal eye field cells, the upregulation of the majority of the EFTF genes orientates towards early retinal progenitor cells (*Lhx2*, *Otx2*, *Pax6*, *Rax*, *Sox2*, and *Six3* but not *Six6*). We suspect that our cells likely finish their differentiation into progenitors later in their development, as suggested by the proliferation tests of isolated D4 RPCs and immunostainings of later-stage organoids (with anti-VSX2, for instance). Determining precisely when the cells acquire a progenitor identity will require cell isolations from several later time points in organoid development. Our data from day 4 cells demonstrate that these early neural progenitors acquired a retinal fate and can be followed since their early commitment.

GSEA analysis of our transcriptomic data shows that synapse formation-related gene sets were among the most upregulated in D4 RPCs compared to stem cells. It is important to note that synapses in mouse retina form between P5 and P14 when all major retinal cell types are differentiated [66]. Our PCA analysis suggests that isolated D4 RPCs have an overall corresponding developmental age younger than E14. We can, therefore, interpret these results either as the activation of very early processes of synapse formation in the layer of RPC/neuroblast cells or that neurotransmitter receptors, for instance, may play a role during neurogenesis. Indeed, classical neurotransmitter signals, like GABA, acetylcholine, glycine, glutamate, and ATP, are well known for modulating the retinal progenitor cell cycle during early retinogenesis [67]. For example, Sholl-Franco and colleagues showed that ATP-activated P2Y_1_ receptors activate the proliferation of retinal progenitor cells via the upregulation of cyclin D1 and downregulation of p27^kip1^ [68]. We previously demonstrated that CHRNB4 (Cholinergic Receptor Nicotinic Beta 4 Subunit) is expressed in early RPCs starting at E15.5 [69] and that the expression is progressively restricted to mature cones during retina development. Although the function has yet to be unraveled, the expression of such a protein family may have a relevant significance for the RPC lineage status.

### 4.5. Possible Applications

The generated cell line enables a variety of applications. Pure cultures of isolated mouse retinal progenitors may shed further light on retinal specification mechanisms. Progenitors undergo this specification during retinal development, giving rise to seven types of terminally differentiated cells in the retina. Simultaneously, the proliferative pattern of the progenitors changes; at the beginning of retinogenesis, most of them divide symmetrically, while later, asymmetrical divisions start to appear and prevail, and, in the end, only symmetrical differentiating divisions occur [70]. However, the molecular underpinnings of these transitions need to be understood. For example, one of the unknown factors is the role of super-enhancers in *Rax* gene control [71]. This cell line may help to pick up the RPCs at these interesting intermediate development time points later than day 4. Next, this cell line might provide a fast output on progenitor or photoreceptor viability in drug screenings on retinal organoids. Similarly, adjustments for organoid production protocols aiming to change the photoreceptor composition can be performed. Organoid-isolated progenitors or photoreceptor precursors could be used to test novel cell-based therapies and help to progress from pre-clinical to clinical studies. Additionally, this cell line would enable the tracing and isolation of RAX-positive cells in non-retinal tissues, such as the pituitary, hypothalamus, and pineal gland, where the RAX protein is a potent regulator [24]. Finally, these stem cells could be used to produce the *Crx-GFP;Rax-mCherry* transgenic mouse line via tetraploid complementation [72]. Once generated, the retinal progenitor development or *Rax* gene regulatory network dynamics could be questioned both in vivo and in vitro.

We show that isolated RAX-positive cells from day 4 organoids proliferate and form colonies when supplemented with growth factors. Although we demonstrated that the day 4 cells commit early to RPC with a proliferating capacity, our next aim is to determine robust culture conditions that allow for long-term culture and the generation of homogeneous retinal organoids. Two recent studies provided examples of the use and analyses of organoid-derived retinal progenitors, which can also be applied to photoreceptor precursors or mature cells. The first one was based on label-free “ghost” cytometry to isolate retinal progenitor cells and form spheroids with retinal epithelium [73]. Their spheroids showed large zones of epithelium, which are suitable for producing retinal grafts for transplantation experiments. Moreover, these spheroid-derived grafts transplanted into a rat model of retinal degeneration showed signs of integration. In the second study, a new method of isolating human early (preneurogenic) retinal progenitors from retinal organoids was described [22]. Additionally, the authors developed a culture medium to expand and cryopreserve retinal progenitors while retaining innate multipotency. The composition of this medium aimed to mimic the environment of the developing retina. As a result, thawed organoid-derived progenitors could be cultured up to 4 passages without losing potency and differentiated to the main retinal cell types, including retinal pigmented epithelium. These studies present valuable methods and approaches to implement the use of RAX-positive mouse retinal progenitors from Crx-GFP;Rax-mCherry retinal organoids in further studies. We will also test the possibility of isolating stem cell-derived mouse progenitors to be cultured, cryopreserved, and rapidly differentiated into retinal cells, according to Gozlan et al. [22]. In conclusion, this mouse cell line may help in the faster undertaking of some biological studies that can then be confirmed with human retinal organoids, which still necessitate a longer period of differentiation and maturation.

## Figures and Tables

**Table 1:** Mouse embryonic fibroblast medium.

Ingredient	Volume	Final Concentration	Producer, Ref.
GMEM	439.5 mL	Not applicable	21710-082, Gibco, Thermo Fisher Scientific, Waltham, MA, USA
Non-essential amino acid (100X)	5 mL	1%	M7145, Sigma-Aldrich, Saint Louis, MO, USA
Sodium Pyruvate (100X)	5 mL	1%	S8636, Sigma-Aldrich, Saint Louis, MO, USA
FBS	50 mL	10%	F7524, Sigma-Aldrich, Saint Louis, MO, USA
2-mercaptoethanol	0.5 mL	0.1%	21985-023, Gibco, Thermo Fisher Scientific, Waltham, MA, USA

**Table 2:** Embryonic stem cell (ES) medium.

Ingredient	Volume	Final Concentration	Producer, Ref.
GMEM	434.5 mL	Not applicable	21710-082, Gibco, Thermo Fisher Scientific, Waltham, MA, USA
Non-essential amino acid (100X)	5 mL	1%	M7145, Sigma-Aldrich, Saint Louis, MO, USA
Sodium Pyruvate (100X)	5 mL	1%	S8636, Sigma-Aldrich, Saint Louis, MO, USA
FBS	5 mL	1%	F7524, Sigma-Aldrich, Saint Louis, MO, USA
KnockOut Serum Replacement	50 mL	10%	10828-028, Thermo Fisher Scientific, Waltham, MA, USA

**Table 3:** ES maintenance medium.

Ingredient	Volume	Final Concentration	Producer, Ref.
ES medium	49.995 mL	Not applicable	N/A
LIF	5 µL	10 ng/mL	300-05, Peprotech, Cranbury, NJ, USA

**Table 4:** Optic vesicle induction medium.

Ingredient	Volume	Final Concentration	Producer, Ref.
GMEM	467 mL	Not applicable	21710-082, Gibco, Thermo Fisher Scientific, Waltham, MA, USA
Non-essential amino acid (100X)	5 mL	1%	M7145, Sigma-Aldrich, Saint Louis, MO, USA
Sodium Pyruvate (100X)	5 mL	1%	S8636, Sigma-Aldrich, Saint Louis, MO, USA
KnockOut Serum Replacement	7.5 mL	1.5%	10828-028, Thermo Fisher Scientific, Waltham, MA, USA
2-mercaptoethanol	0.5 mL	0.1%	21985-023, Gibco, Thermo Fisher Scientific, Waltham, MA, USA
N2 (100X)	5 mL	1%	17502048, Thermo Fisher Scientific, Waltham, MA, USA
B27 without vitamin A (50X)	10 mL	2%	12587010, Thermo Fisher Scientific, Waltham, MA, USA

**Table 5:** Optic-cup induction medium.

Ingredient	Volume	Final Concentration	Producer, Ref.
DMEM/F12-GlutaMAX	49.5 mL	Not applicable	31331028, Thermo Fisher Scientific, Waltham, MA, USA
N2 (100X)	0.5 mL	1%	17502048, Thermo Fisher Scientific, Waltham, MA, USA

**Table 6:** Retinal maturation medium I.

Ingredient	Volume	Final Concentration	Producer, Ref.
DMEM/F12-GlutaMAX	47.5 mL	Not applicable	31331028, Thermo Fisher Scientific, Waltham, MA, USA
N2 (100X)	0.5 mL	1%	17502048, Thermo Fisher Scientific, Waltham, MA, USA
B27 (50X)	2 mL	4%	17504044, Thermo Fisher Scientific, Waltham, MA, USA

**Table 7:** Retinal Maturation Medium II.

Ingredient	Volume	Final Concentration	Producer, Ref.
DMEM/F12-GlutaMAX	48.95 mL	Not applicable	31331028, Thermo Fisher Scientific, Waltham, MA, USA
Taurine	50 µL of 100 mM stock	1 mM	T0625-10G, Sigma-Aldrich, Saint Louis, MO, USA
B27 (50X)	2 mL	4%	17504044, Thermo Fisher Scientific, Waltham, MA, USA

**Table 8:** Characteristics of 5 single-guide RNA pairs and prospective double-strand DNA breaks in the vicinity of the *Rax* gene exon three stop codon, proposed by CHOPCHOP web tool. Nucleotide sequences of two guide RNAs with PAM are shown in blue and red color. *sgRNA* pair 105 was selected for gene editing.

Rank	Target Sequence	Genomic Location	Off-Targets				sgRNAs Offset	Double-Strand Break Overhang	Depth of Exon-Coding Region Invasion by Single-Strand Break
			0	1	2	3			
23	GCGCCTCTAGAGGGCTTGCCAGGGCTTTCCGATGGCCTGGCTGTGCTCTTTGGCCTTC	Chr18:65934998	0/0	0/0	0/0	0/2	−58 bp	3′, 24 bp	11 amino acids
97	CCACCAGGCTGGGCTGGGTGCACACAGGGCTCGCAGCAACTCCGCAGCGCCTCTAGAGGGCTTGCCAGG	Chr18:65934952	0/0	0/0	1/0	6/0	23 bp	5′, 57 bp	3 amino acids
105	CCAGGCTGGGCTGGGTGCACACAGGGCTCGCAGCAACTCCGCAGCGCCTCTAGAGGGCTTGCCAGG	Chr18:65934955	0/0	0/0	0/0	12/0	20 bp	5′, 54 bp	3 amino acids
108	CCTCTAGAGGGCTTGCCAGGGCTTTCCGATGGCCTGGATGTGCTCTTTGGCCTTCAGG	Chr18:65935001	0/0	0/0	0/0	6/7	12 bp	5′, 46 bp	16 amino acids
110	GGCTGGGCTGGGTGCACACAGGGCTCGCAACTCCGCAGCGCCTCTAGAGGGCTTGCCAGGGCT	Chr18:65934958	0/0	0/0	0/0	9/6	−66 bp	3′, 32 bp	0 amino acids

## Data Availability

Raw RNA sequencing data used for Figure 7 and Supplementary Materials was deposited in the National Center for Biotechnology Information (NCBI) Sequence Read Archive and is accessible with BioProject ID PRJNA1219972. The mass spectrometry proteomics data used for Figure 8 have been deposited to the ProteomeXchange Consortium via the PRIDE [74] partner repository with the dataset identifier PXD039309.

## Supplementary Materials

The following supporting information can be downloaded at: https://www.mdpi.com/article/10.3390/cells14040252/s1, Figure S1: Top-20 enriched gene sets in WT PR and mESC in comparisons of D4 RPC with wild-type mature photoreceptors (PR WT) and mESC, respectively; Figure S2: Expression of endogenous Crx-GFP and Rax-mCherry on day 19 of retinal organoid development using mESC cl. 1-2-7; Figure S3: Immunolabeling of embryoid bodies with antibodies against main markers of three germ layers—beta-tubulin 3/TUJ1 (ectoderm marker), smooth muscle actin (SMA, mesoderm marker, and alpha-fetoprotein (AFP, endoderm marker). Scale–100 µm; Table S1: Top-20 enriched genes in D4 RPC in comparison “D4 RPC vs. PR WT”; Table S2: Top-20 enriched genes in wild-type mature photoreceptors in comparison “D4 RPC vs. PR WT”.
