# Supplementary material for: Generation of a Double Reporter mES Cell Line to Simultaneously Trace the Generation of Retinal Progenitors and Photoreceptors
Source: Cells. 2025 Feb 10;14(4):252. doi: 10.3390/cells14040252 (PMC11854395; doi:10.3390/cells14040252)
Supplement: Supplementary file 1 [file cells-14-00252-s001.zip › cells-3170820-supplementary.pdf]

cells

MDPI

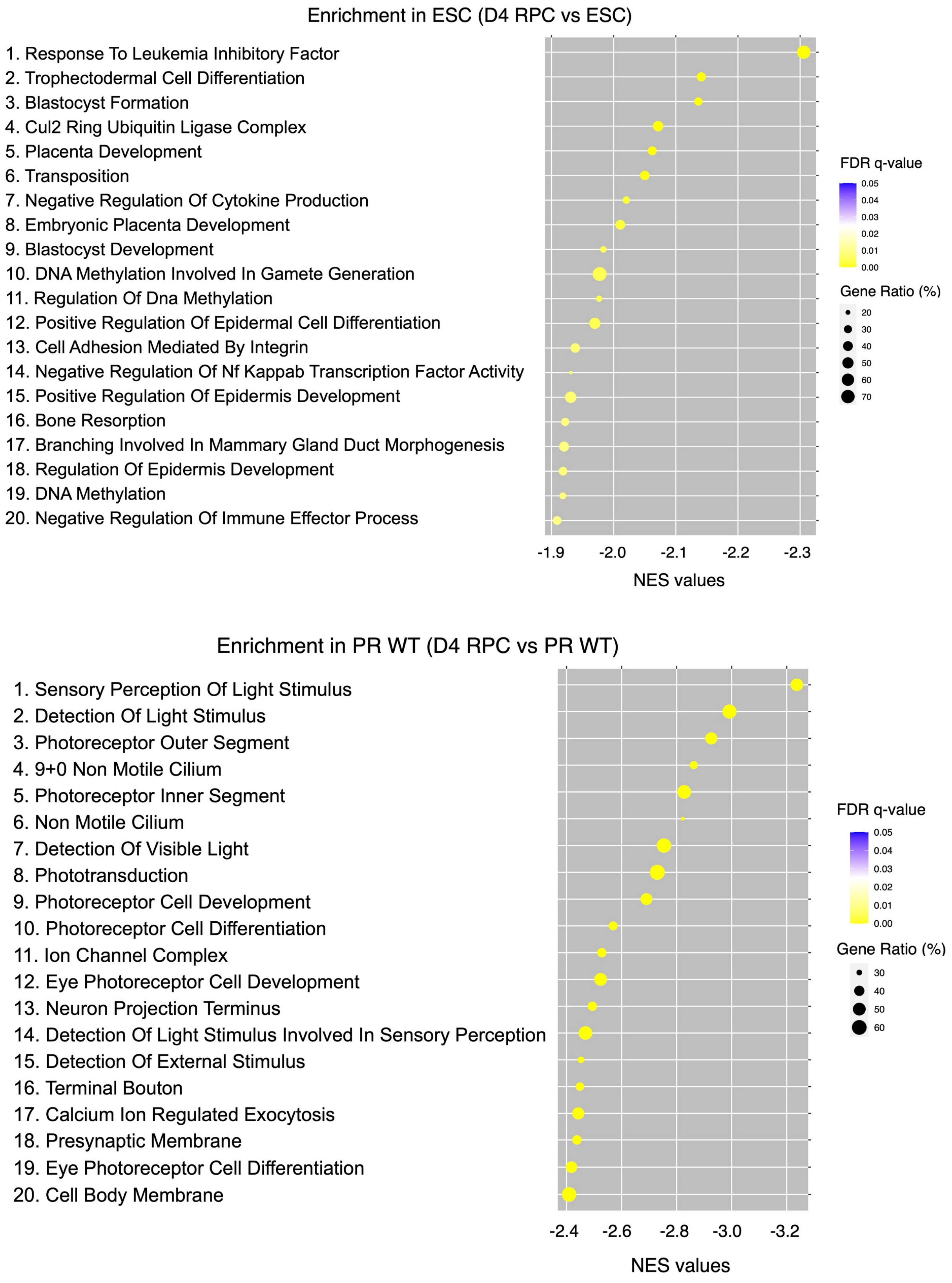

Figure S1. Top-20 enriched gene sets in WT PR and mESC in comparisons of D4 RPC with wild-type mature photoreceptors (PR WT) and mESC, respectively.

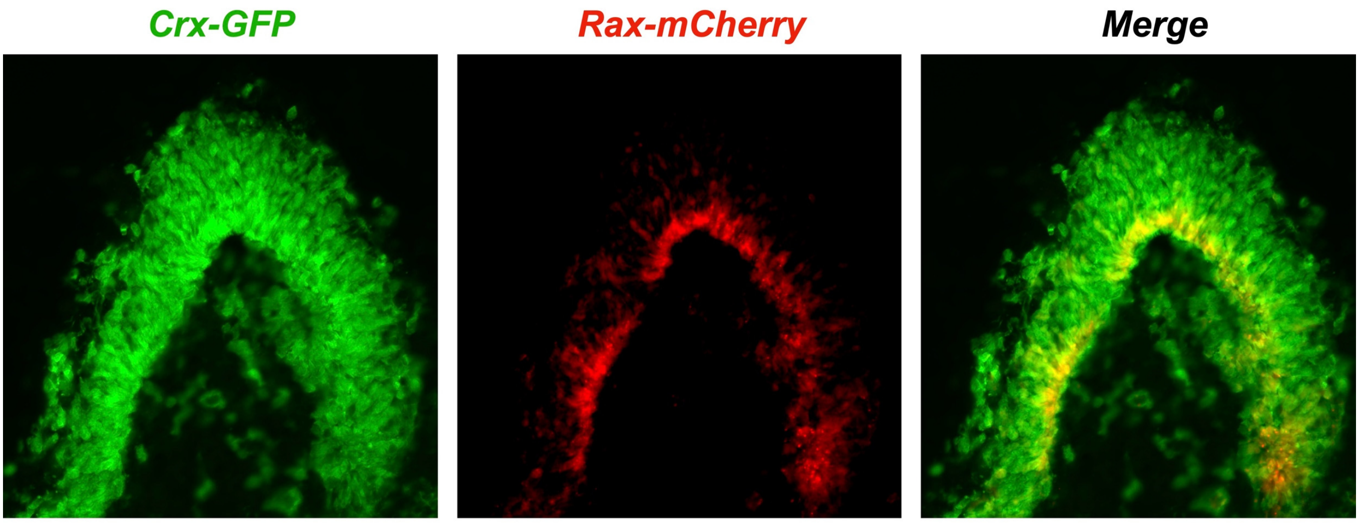

Figure S2. Expression of endogenous Crx-GFP and Rax-mCherry on day 19 of retinal organoid development using mESC cl. 1-2-7.

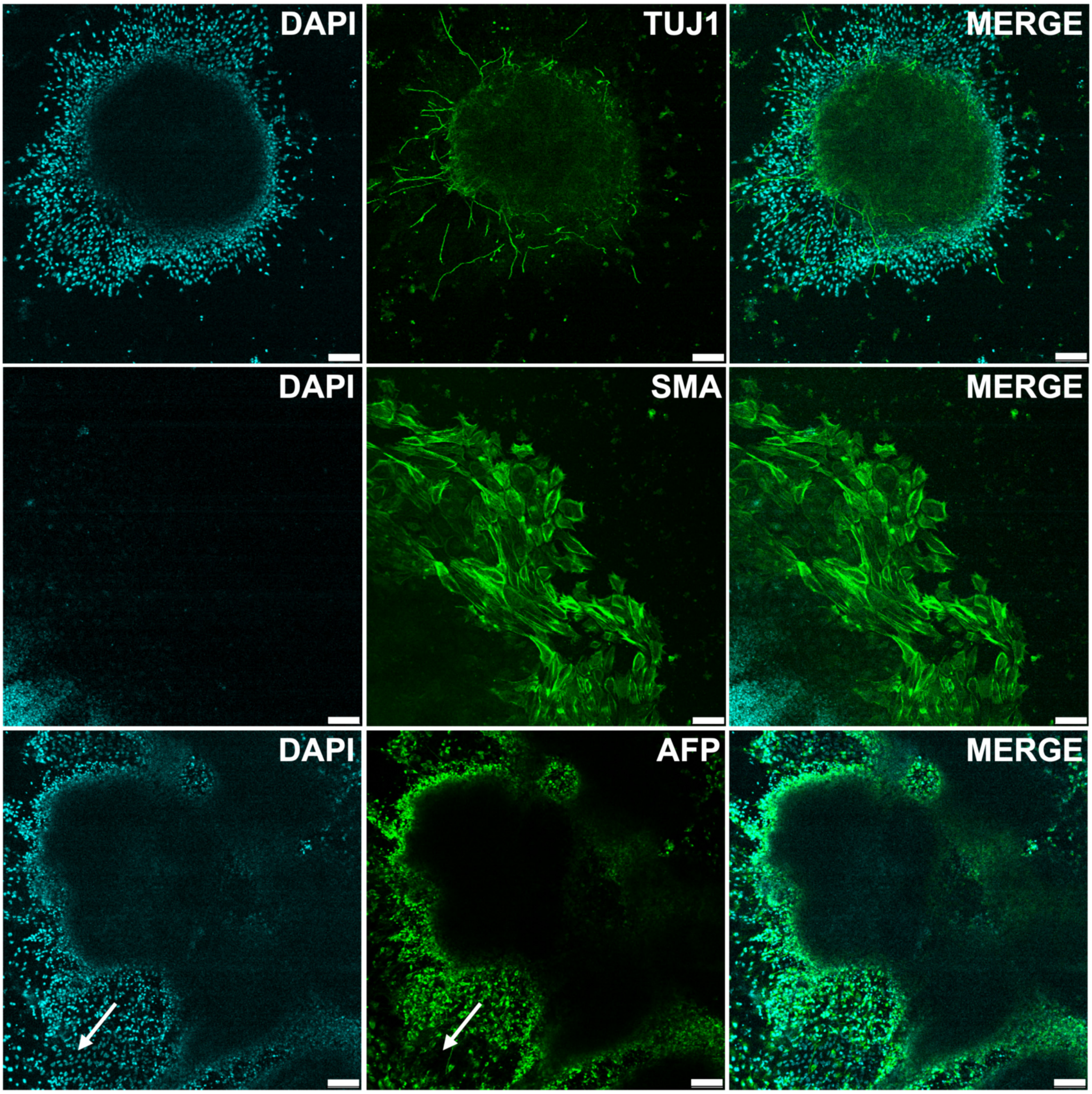

Figure S3. Immunolabeling of embryoid bodies with antibodies against main markers of three germ layers — beta-tubulin 3/TUJ1 (ectoderm marker), smooth muscle actin (SMA, mesoderm marker, and alpha-fetoprotein (AFP, endoderm marker). Scale – 100 µm.

1	Rpl10-ps3	ribosomal protein L10, pseudogene 3 [Source:MGI Symbol;Acc:MGI:3704336]
2	Sox21	SRY (sex determining region Y)-box 21 [Source:MGI Symbol;Acc:MGI:2654070]
3	Gm10123	predicted pseudogene 10123 [Source:MGI Symbol;Acc:MGI:3704342]
4	Mki67	antigen identified by monoclonal antibody Ki 67 [Source:MGI Symbol;Acc:MGI:106035]
5	Ccnd2	cyclin D2 [Source:MGI Symbol;Acc:MGI:88314]
6	Lin28b	lin-28 homolog B [Source:MGI Symbol;Acc:MGI:3584032]
7	Sfrp1	secreted frizzled-related protein 1 [Source:MGI Symbol;Acc:MGI:892014]
8	Sox1	SRY (sex determining region Y)-box 1 [Source:MGI Symbol;Acc:MGI:98357]
9	Hmga2	high mobility group AT-hook 2 [Source:MGI Symbol;Acc:MGI:101761]
10	Tpt1-ps3	tumor protein, translationally-controlled, pseudogene 3 [Source:MGI Symbol;Acc:MGI:2664997]
11	Trim71	tripartite motif-containing 71 [Source:MGI Symbol;Acc:MGI:2685973]
12	Rps3a2	ribosomal protein S3A2 [Source:MGI Symbol;Acc:MGI:3642853]
13	Lhx5	LIM homeobox protein 5 [Source:MGI Symbol;Acc:MGI:107792]
14	Arx	aristaless related homeobox [Source:MGI Symbol;Acc:MGI:1097716]
15	Top2a	topoisomerase (DNA) II alpha [Source:MGI Symbol;Acc:MGI:98790]
16	Rps3a3	ribosomal protein S3A3 [Source:MGI Symbol;Acc:MGI:3643406]
17	Gm12671	predicted gene 12671 [Source:MGI Symbol;Acc:MGI:3651684]
18	Fndc3c1	fibronectin type III domain containing 3C1 [Source:MGI Symbol;Acc:MGI:2685630]
19	Bub1	BUB1, mitotic checkpoint serine/threonine kinase [Source:MGI Symbol;Acc:MGI:1100510]
20	Kif15	kinesin family member 15 [Source:MGI Symbol;Acc:MGI:1098258]

Table S1. Top-20 enriched genes in D4 RPC in comparison “D4 RPC vs PR WT”.

1	Prph2	peripherin 2 [Source:MGI Symbol;Acc:MGI:102791]
2	Rcvrn	recoverin [Source:MGI Symbol;Acc:MGI:97883]
3	Rbp3	retinol binding protein 3, interstitial [Source:MGI Symbol;Acc:MGI:97878]
4	Rho	rhodopsin [Source:MGI Symbol;Acc:MGI:97914]
5	Nrl	neural retina leucine zipper gene [Source:MGI Symbol;Acc:MGI:102567]
6	Nr2e3	nuclear receptor subfamily 2, group E, member 3 [Source:MGI Symbol;Acc:MGI:1346317]
7	Grm6	glutamate receptor, metabotropic 6 [Source:MGI Symbol;Acc:MGI:1351343]
8	Pdc	phosducin [Source:MGI Symbol;Acc:MGI:98090]
9	Scgn	secretagogin, EF-hand calcium binding protein [Source:MGI Symbol;Acc:MGI:2384873]
10	Slc17a7	solute carrier family 17 (sodium-dependent inorganic phosphate cotransporter), member 7 [Source:MGI Symbol;Acc:MGI:1920211]
11	Pde6h	phosphodiesterase 6H, cGMP-specific, cone, gamma [Source:MGI Symbol;Acc:MGI:1925850]
12	Pde6g	phosphodiesterase 6G, cGMP-specific, rod, gamma [Source:MGI Symbol;Acc:MGI:97526]
13	Gabrr1	gamma-aminobutyric acid (GABA) C receptor, subunit rho 1 [Source:MGI Symbol;Acc:MGI:95625]
14	Pde6b	phosphodiesterase 6B, cGMP, rod receptor, beta polypeptide [Source:MGI Symbol;Acc:MGI:97525]
15	Rp1l1	retinitis pigmentosa 1 homolog like 1 [Source:MGI Symbol;Acc:MGI:2384303]
16	Vsx2	visual system homeobox 2 [Source:MGI Symbol;Acc:MGI:88401]
17	Gm10288	predicted gene 10288 [Source:MGI Symbol;Acc:MGI:3704227]
18	Gsg1	germ cell associated 1 [Source:MGI Symbol;Acc:MGI:1194499]
19	Opn1sw	opsin 1 (cone pigments), short-wave-sensitive (color blindness, tritan) [Source:MGI Symbol;Acc:MGI:99438]
20	Gm5537	predicted gene 5537 [Source:MGI Symbol;Acc:MGI:3643179]

Table S2. Top-20 enriched genes in wild-type mature photoreceptors in comparison "D4 RPC vs PR WT".
